# Experimental data on the relationship between dyes sensitizers and wavelength during the photocatalytic degradation of diclofenac

**DOI:** 10.1016/j.dib.2019.104370

**Published:** 2019-08-08

**Authors:** J. Diaz-Angulo, J. Porras, M. Mueses, R.A. Torres-Palma, F. Machuca-Martinez

**Affiliations:** a Grupo GAOX, Escuela de Ingeniería Química, Universidad del Valle, Cali, Colombia; b Grupo de Investigaciones Biomédicas Uniremington, Facultad de Ciencias de la Salud, Corporación Universitaria Remington (Uniremington), Calle 51 No. 51-27, Medellín, Colombia; c Photocatalysis and Solar Photoreactors Engineering, Department of Chemical Engineering, Universidad de Cartagena, Cartagena, Colombia; d Grupo de Investigación en Remediación Ambiental y Biocatálisis (GIRAB), Instituto de Química, Facultad de Ciencias Exactas y Naturales, Universidad de Antioquia UdeA, Calle 70 No. 52-21, Medellín, Colombia

**Keywords:** Organic dyes, Dye-sensitized, Perinaphtenone, Eosin-Y, TiO_2_, Sensitization, Visible light, Photocatalysis

## Abstract

Sensitizers are being used to improve the photocatalytic activity of semiconductors in the visible light region of the solar spectrum. Different types of dyes are reported as sensitizer agents, such as ruthenium complex molecules, porphyrins and Pt complexes, which are critically assessed because they are hazardous substance. Therefore, it is necessary to replace these compounds with safer sensitizer like organic dyes. This work evaluated the photocatalytic degradation of diclofenac using two different types of organic dyes (Perinaphtenone and Eosin-Y) as sensitizer agents. The catalyst concentration [0.15; 0.35 g/l], source of light (UVA – Vis) and type of dye were evaluated. The data obtained can be useful to classify organic dyes that could be employees as sensitizers and which is the wavelength more adequate to use as an energy source. The Kapp for the reaction has values between 1*10^−3^ to 5*10^−3^ min^−1^ for UVA, 3*10^−4^ to 3*10^−3^ min^−1^ for Vis and 2*10^−3^ to 6*10^−3^ min^−1^ for UV–Vis.

**Table:** Specifications table

Subject area	Chemical engineering
More specific subject are	Advanced oxidation process
Type of data	Figure and table
How data was acquired	Data were obtained by UV–vis spectrophotometry and high liquid performance chromatography
Data format	Analyzed
Experimental factors	All experimental tests were carried out to laboratory-scale in a device equipped with six fluorescent tubes interchangeable.
Experimental features	The experimental data were obtained to evaluate the diclofenac degradation by dye sensitized of TiO_2_ considering the dye type and the energy source at different catalyst loading.
Data source location	GAOX, Universidad del Valle, Cali, Colombia.
Data accessibility	The data is found only in this article.
Related research article	J. Diaz-Angulo, I. Gomez-Bonilla, C. Jimenez-Tohapanta, M. Mueses, M. Pinzon, F. Machuca-Martinez, Visible-light activation of TiO2 by dye-sensitization for degradation of pharmaceutical compounds, Photochem. Photobiol. Sci. 18 (2019) 897–904. https://doi.org/10.1039/c8pp00270c

**Table:** 

**Value of the data** • Data obtained show that dye-sensitized of TiO_2_ is an appropriate technique to improve the diclofenac degradation under the adequate energy source. • Data can be used to compare different dyes to sensitized TiO_2_. • Data could be useful for scaling up of the sensitized process. • Data may be useful in future research on dye sensitization process.

## 1 Data

**Fig. 1:**
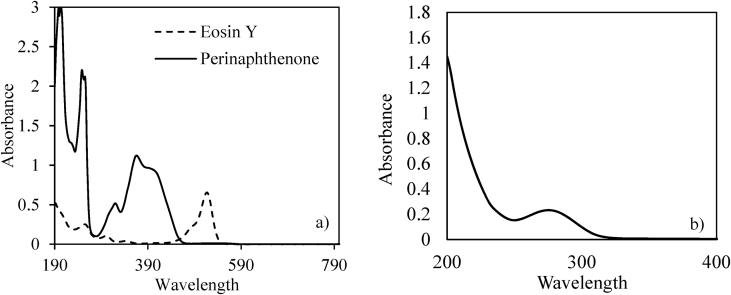
Absorption spectra of the compound used. (a) organic dyes [Dye] = 4 mg/L. (b) Diclofenac [30 mg/L].

**Fig. 2:**
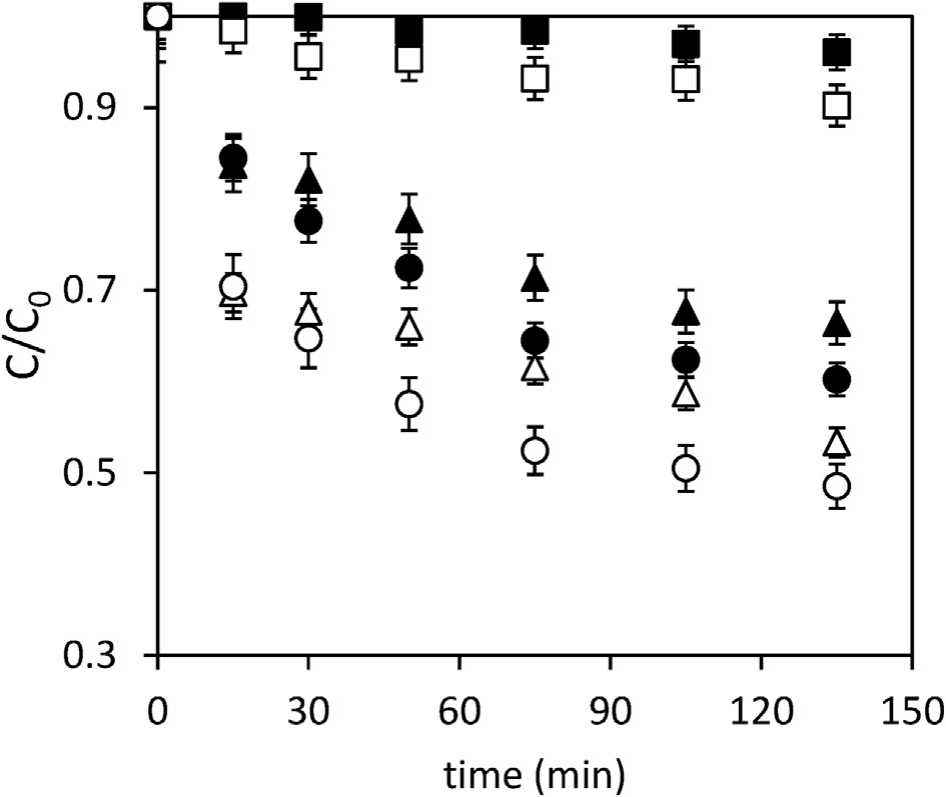
Diclofenac degradation under visible light. [TiO_2_] = 0.15 g/L; [DCF] = 30 mg/L. [Dye] = 4 mg/L. (■) photolysis, (□) TiO_2_-DCF, (▲) Ph-DCF, (Δ) TiO_2_-Ph-DCF, (●) Ey –DCF and (○) TiO_2_-Ey-DCF.

**Fig. 3:**
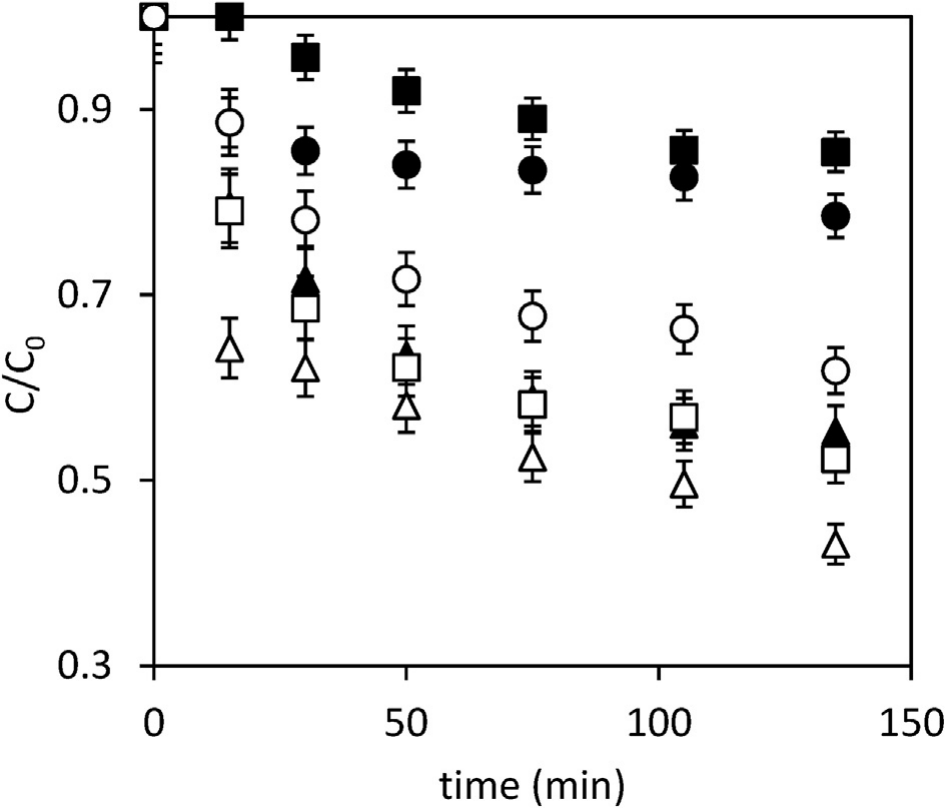
Diclofenac degradation under UVA light, [TiO_2_] = 0.15 g/L; [DCF] = 30 mg/L. [Dye] = 4 mg/L. (■) photolysis, (□) TiO_2_-DCF, (▲) Ph-DCF, (Δ) TiO_2_-Ph-DCF, (●) Ey –DCF and (○) TiO_2_-Ey-DCF.

**Fig. 4:**
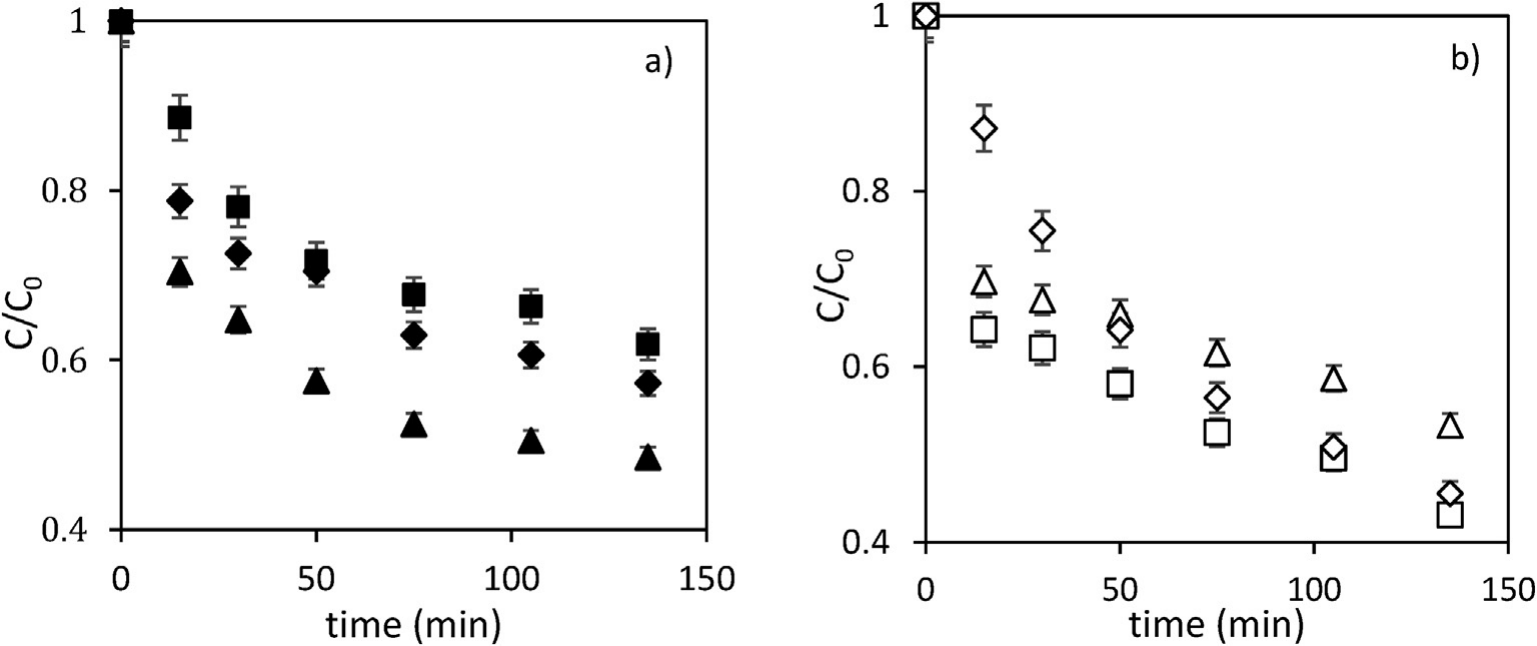
Comparison of diclofenac degradation by dye-sensitized of TiO_2_ under different sources of energy, [TiO_2_] = 0.15 g/L; [DCF] = 30 mg/L. [Dye] = 4 mg/L; (a) Eosin Y (■) TiO_2_-DCF-UVA, (♦) TiO_2_-DCF-Vis (▲) TiO_2_-DCF-UVA-Vis and (b) Perinaphtenone (□)TiO_2_-DCF-UVA, (◊) TiO_2_-DCF-Vis and (Δ) TiO_2_-DCF- UVA-Vis.

**Fig. 5:**
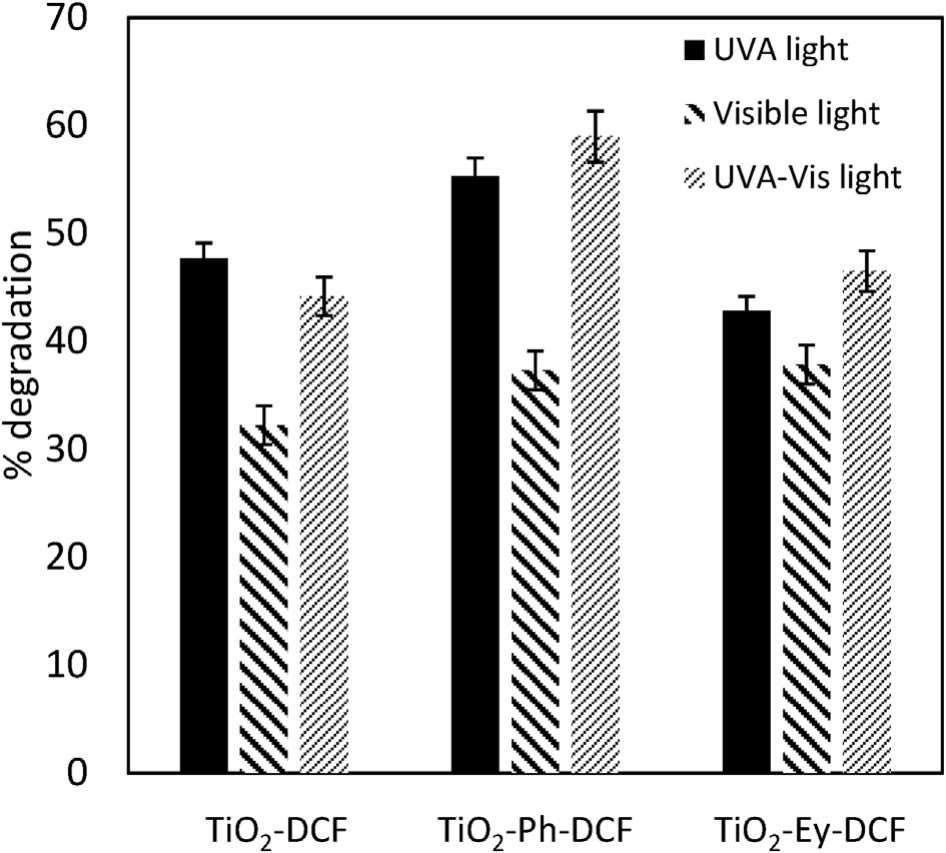
Comparison of diclofenac degradation by dye-sensitized of TiO_2_ under different sources of energy, [TiO_2_] = 0.35 g/L; [DCF] = 30 mg/L.

Data present in this work describes the diclofenac degradation by photocatalysis, photosensitized oxidation, and dye-sensitized of TiO_2_ using organic dyes that are a promising technique because of great result has been obtained for the degradation of several contaminants [1], [2], [3], [4]. Table 1 shows the properties of all compounds. Fig. 1 presents the UV/vis spectra of the Perinaphtenone (Ph) and Eosin-Y (Ey) dyes used as a sensitizer agent and diclofenac (DFC), this allows knowing the maximum wavelength which absorbs energy each dye and the compound. Fig. 2 and Fig. 3 illustrate the variation of diclofenac (DFC) degradation according to the energy source Visible and UVA light, respectively. Tests of photolysis, photocatalysis and photosensitized oxidation were performed in order to identify synergies. Finally, Fig. 4 and Fig. 5 show the influence of each source of energy for dye-sensitized of TiO_2_ for 0.35 gL^−1^ of catalyst concentration. In the supplementary material the raw data of Fig. 2, Fig. 3, Fig. 4 can be seen.

**Table 1:** Properties of the compound used.

Molecular structure	Name and nomenclature	λ (nm)	Molecular formula	Molecular weight (gmol^−1^)	Melting point (°C)	Water solubility (mgmL^−1^) (at 25 °C)
	Perinaphtenone (Ph)	368	C_13_H_8_O	*180.20*	*153–156*	*not very* soluble
	Eosin-Y (Ey)	518	C_20_H_6_Br_4_Na_2_O_5_	*647.89*	*300*	*Very soluble*
	Diclofenac (DFC)	276	C_14_H_10_Cl_2_NNaO_2_	*318.13*	*156–158*	*soluble*

Table 2 shows the experimental conditions and the diclofenac degradation by photolysis, photocalysis and dye sensitization process using Perinaphtenone (Ph) and Eosin-Y (Ey) as sensitizers.

**Table 2:** Experimental conditions and diclofenac degradation by photolysis, photocalysis and dye sensitization process.

	[TiO_2_] = 0.15 g/L						
	UVA		Visible		UVA+Visible		
	[DCF]_final_ mg/L	% deg	[DCF]_final_ mg/L	% deg	[DCF]_final_ mg/L	% deg	pH (±0.3)
Photolysis	25.6	14.7	28.8	4.0	–		6.8
TiO_2_-DCF	15.7	47.7	27.0	10.0	18.9	37.0	7.2
Ph-DCF	16.6	44.7	19.9	33.7	13.9	53.7	7.3
EY-DCF	23.6	21.3	18.0	40.0	21.1	29.7	7.1
TiO_2_-Ph-DCF	13.0	56.7	16.0	46.7	13.6	54.7	7.2
TiO_2_-EY-DCF	18.6	38.0	14.6	51.3	17.2	42.7	7.2
[TiO_2_] = 0.35 g/L							
TiO_2_-DCF	15.0	47.7	20.3	32.2	16.7	44.2	7.1
TiO_2_-Ph-DCF	13.4	55.3	18.8	37.3	12.3	59.0	7.4
TiO_2_-EY-DCF	17.1	42.9	18.6	37.8	16.04	46.5	7.4

Table 3 shows the Kapp for the degradation of DFC by TiO_2_ using Perinaphtenone (Ph) and Eosin-Y (Ey) as sensitizers.

**Table 3:** Kapp for diclofenac degradation by photolysis, photocatalysis and dye sensitizartion process os TiO_2._

	UVA (Kapp min^−1^)	Visible (Kapp min^−1^)	UVA+Visible (Kapp min^−1^)
Photolysis	1 x 10^−3^ ± 7 x 10^−5^	3 x 10^−4^ ± 2 x10^−5^	–
TiO_2_-DCF	4 x 10^−3^ ± 2 x10^−4^	7 x 10^−4^ ± 4 x10^−5^	–
Ph-DCF	4 x 10^−3^ ± 2 x10^−4^	3 x 10^−3^ ± 1 x10^−4^	6 x 10^−3^ ± 2 x 10^−5^
EY-DCF	1 x 10^−3^ ± 7 x 10^−5^	4 x 10^−3^ ± 2 x10^−4^	2 x 10^−3^ ± 4 x 10^−5^
TiO_2_-Ph-DCF	5 x 10^−3^ ± 3 x10^−4^	4 x 10^−3^ ± 2 x 10^−4^	6 x 10^−3^ ± 1 x 10^−4^
TiO_2_-EY-DCF	3 x 10^−3^ ± 1 x10^−4^	5 x 10^−3^ ± 3 x 10^−4^	4 x 10^−3^ ± 2 x 10^−4^

## 2 Experimental design, materials, and methods

### 2.1 Material

Diclofenac sodium (CAS 15307-79-6, Sigma Aldrich), Perinaphthenone (97% CAS 548-39-0, Sigma Aldrich) and Eosin Y (EY; Color index (C.I.) No. 45380; Fisher Chemical - ChemAlert) were used as received. The catalyst TiO2 Degussa P-25 was obtained from Degussa Corporation (99.5% Evonik. No. CAS 13463-67-7, 80% anatase and 20% rutile crystalline phases; a specific surface area of 50 m^2^ g^−1^). Additionally, acetonitrile (Sigma Aldrich, 99.99% analytical grade) and formic acid (Sigma Aldrich, reagent grade ≥95%) were used for the mobile phase. For experimental tests water purified by a Millipore Milli-Q device was used.

### 2.2 Reactive system

**Fig. 6:**
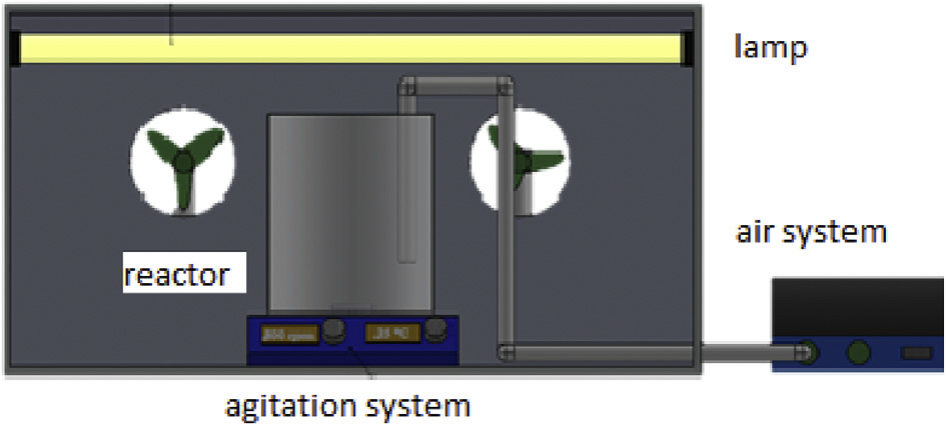
Experimental scheme of the reactive system.

Photocatalytic reactions were performance is a batch reactor (Pyrex glass bottle) illuminated from the top in a device equipped with six fluorescent tubes interchangeable. UVA light was emitted by tubes TLAD 30W05 Philips with wavelengths between 300 and 450 nm and a maximum at 365 nm [5], [6]. Visible lamps Sylvania F30W, emitting above of 400 nm were used. The temperature was kept constant to 27 ± 2 °C and air was supplied to each system with a flow rate of 2 L/min to maintain the concentration of dissolved oxygen constant. Fig. 6 shows a scheme of the device.

### 2.3 Experimental

DCF solution was prepared at 30 mg/L. The reaction volume was 0.25 L. TiO_2_ concentrations and the dye sensitizer (Eosin Y or Perinaphthenone) were added simultaneously [1], [7]. Subsequently, the reactive system was stirred magnetically in darkness for 30 minutes in order to promote the adsorption of DCF and the sensitizer onto the catalyst surface [8], [9]. After the adsorption period, the slurry was irradiated for a period of 150 minutes. Aliquots (2 ml) were taken at different intervals to perform analyzes. All tests were repeated three times to ensure the data reproducibility.

### 2.4 Analytical techniques

High-resolution liquid chromatography (HPLC) Thermo scientific ultimate 3000 with a diode array detector (DAD) was used to determine the concentration of DCF using a LiChrosphere® 100 RP-18 column (5μm). A mixture of 35% water (10mM formic acid) and 65% acetonitrile operated in isocratic mode was used as mobile phase at a flow rate of 0.85 mlmin^−1^. To obtain the dye spectra UV–Vis 1800 spectrophotometer was used.
