# Supplementary material for: Experimental data on the relationship between dyes sensitizers and wavelength during the photocatalytic degradation of diclofenac
Source: Data Brief. 2019 Aug 8;25:104370. doi: 10.1016/j.dib.2019.104370 (PMC6706764; doi:10.1016/j.dib.2019.104370)
Supplement: Multimedia component 1 [file mmc1.docx]

EXPERIMENTAL DATA ON THE RELATIONSHIP BETWEEN DYES SENSITIZERS AND WAVELENGTH DURING THE PHOTOCATALYTIC DEGRADATION OF DICLOFENAC

Diaz-Angulo, J.^1^ Porras, J^2^. Mueses, M^3^. Torres-Palma, R^4^. Machuca-Martinez, F.^1*^

(1) GAOX Group, School of Chemical Engineering, Universidad del Valle, Cali, Colombia.

(2) Grupo de investigaciones biomédicas, Uniremington, Medellín, Colombia.

(3) Photocatalysis and Solar Photoreactors Engineering, Department of Chemical Engineering, Universidad de Cartagena, Cartagena, Colombia.

(4) Grupo GIRAB, Química, Facultad de Ciencias Exactas y Naturales, Universidad de Antioquia UdeA, Calle 70 No. 52-21, Medellín, Colombia.

*Corresponding author: [fiderman.machuca@correounivalle.edu.co](mailto:fiderman.machuca@correounivalle.edu.co)

Supplementary material

Table S1. Experimental raw data of the decrease in the diclofenac concentration by photolysis, photocatalysis and dye-sensitization process under visible light.

| time | photolysis | TiO_2_-DCF | Ph-DCF | Ey-DCF | TiO_2_-Ph-DCF | TiO_2_-Ey-DCF |
| --- | --- | --- | --- | --- | --- | --- |
| 0 | 30.1 | 29.8 | 30.2 | 30.0 | 30.0 | 30.2 |
| 15 | 29.8 | 29.5 | 25.1 | 25.3 | 20.9 | 21.1 |
| 30 | 30.0 | 28.7 | 24.6 | 23.3 | 20.3 | 19.4 |
| 50 | 29.5 | 28.6 | 23.3 | 21.7 | 19.8 | 17.3 |
| 75 | 29.5 | 28.0 | 21.4 | 19.3 | 18.5 | 15.7 |
| 105 | 29.1 | 27.9 | 20.3 | 18.7 | 17.6 | 15.1 |
| 135 | 28.8 | 27.1 | 19.9 | 18.1 | 16.0 | 14.6 |

Table S2. Experimental raw data of the decrease in the diclofenac concentration by photolysis, photocatalysis and dye-sensitization process under UVA light.

| time | photolysis | TiO2-DCF | Ph-DCF | Ey-DCF | TiO_2_-Ph-DCF | TiO_2_-Ey-DCF |
| --- | --- | --- | --- | --- | --- | --- |
| 0 | 30.2 | 30.0 | 30.1 | 30.4 | 30.0 | 30.3 |
| 15 | 30.0 | 23.7 | 23.9 | 26.6 | 19.3 | 26.6 |
| 30 | 28.7 | 20.6 | 21.5 | 25.7 | 18.6 | 23.4 |
| 50 | 27.6 | 18.7 | 19.1 | 25.2 | 17.4 | 21.5 |
| 75 | 26.7 | 17.5 | 17.6 | 25.0 | 15.7 | 20.3 |
| 105 | 25.7 | 17.0 | 16.8 | 24.8 | 14.9 | 19.9 |
| 135 | 25.6 | 15.7 | 16.6 | 23.6 | 12.9 | 18.6 |

Table S3. Experimental raw data of the decrease in the diclofenac concentration by dye-sensitization process under UVA+Visible light.

| time | Ph-DCF | Ey-DCF | TiO_2_-Ph-DCF | TiO_2_-Ey-DCF |
| --- | --- | --- | --- | --- |
| 0 | 30.1 | 30.5 | 30.2 | 30.3 |
| 15 | 28.8 | 24.2 | 26.2 | 23.6 |
| 30 | 23.4 | 22.4 | 22.6 | 21.8 |
| 50 | 19.8 | 22.5 | 19.2 | 21.1 |
| 75 | 17.0 | 21.8 | 16.9 | 18.9 |
| 105 | 16.3 | 20.8 | 15.3 | 18.2 |
| 135 | 13.9 | 21.2 | 13.7 | 17.2 |
